# *In situ* soil moisture and temperature network in genhe watershed and saihanba area in China

**DOI:** 10.1016/j.dib.2020.105693

**Published:** 2020-05-19

**Authors:** Lingmei Jiang, Jian Wang, Huizhen Cui, Gongxue Wang, Tianjie Zhao, Shaojie Zhao, Linna Chai, Xiaojing Liu, Jianwei Yang

**Affiliations:** a State Key Laboratory of Remote Sensing Science, Jointly Sponsored by Beijing Normal University and Aerospace Information Research Institute of Chinese Academy of Sciences, Faculty of Geographical Science, Beijing Normal University, Beijing 100875, China; b State Key Laboratory of Remote Sensing Science, Aerospace Information Research Institute, Chinese Academy of Sciences, Beijing 100101, China; c State Key Laboratory of Earth Surface Processes and Resource Ecology, Faculty of Geographical Science, Beijing Normal University, Beijing 100875, China

**Keywords:** Soil moisture, Soil temperature, Genhe watershed, Saihanba area, China

## Abstract

The dataset presented in this article is related to the work “Evaluation and Analysis of SMAP, AMSR2, and MEaSUREs Freeze/Thaw Products in China [1]”. Soil moisture and temperature are important variables of land-atmosphere energy exchange, monitoring vegetation growth, predicting drought disasters and climate and hydrological modelling [2], [3], [4], [5], [6]. This work provides detailed information on *in situ* soil moisture and temperature data network established in the Genhe watershed and Saihanba area in China, respectively. The Genhe watershed represents the complex surface heterogeneity in Northeast China. Therefore, data from 22 *in situ* sites were established in the Genhe watershed since March 2016 to improve the dynamic analysis and modeling of remotely sensed information for complex land surfaces. Saihanba is currently China's largest manmade forest and has a unique alpine wetland and a complete aquatic ecosystem. There are 29 *in situ* sites deployed in Saihanba since August 2018 for studying the cold temperate continental monsoon climate and estimating forest carbon storage capacity and carbon emissions from manmade forests. Soil temperature and permittivity data in the network were measured using ECH2O EC-5TM probes (Decagon Devices, Inc., Washington, USA, https://www.metergroup.com/) and XingShiTu (XST) probes (BEIJING XST Co., Ltd., www.xingshitu.com) every 30 min at depths of 3, 5, and 10 cm for the Genhe watershed continuous automatic observation network, and depths of 5 and 10 cm for the Saihanba continuous automatic observation network. In the Genhe watershed, soil moisture and soil temperature data in the network were automatically collected using the EM50 data collection system. The Saihanba area has the XST data collection system to record soil temperature and permittivity. The permittivity data collected with the XST data collector were transformed to soil moisture data (volumetric water content) based on the formula developed by [7]. The datasets of the Genhe watershed and Saihanba area consist of raw data acquired by the data collector and processed data of soil moisture and temperature. The Saihanba dataset also includes the calibration data based on soil texture. The result of temporal variations analysis in observed data in the Genhe Watershed and the processing in observed data in the saihanba area show that the long-term *in situ* soil moisture and temperature datasets can be used for the validation/calibration and improvement of the soil moisture and soil freeze/thaw algorithm.

## Highlights

• Soil moisture, temperature, permittivity data in Genhe and Saihanba were collected.
• Data were collected for dynamic analysis and modeling complex land surfaces.
• Data can be used to validate the soil moisture and soil freeze/thaw algorithm.

Specifications table

**Table:** 

Subject	Earth-Surface Processes
Specific subject area	Soil moisture and temperature, Remote Sensing, Validation.
Type of data	Tables, figures. The data are stored in Microsoft Excel format in a zip package.
How data were acquired	Soil temperature and permittivity were automatically measured using 5TM and XST probes. Processed data were obtained using MATLAB software processing tool.
Data format	Raw and processed.
Parameters for data collection	Soil moisture and temperature at the depths of 3, 5, and 10 cm for the Genhe watershed and 5 and 10 cm for the Saihanba area.
Description of data collection	Soil moisture (m^3^/m^3^) and temperature (°C) data were collected and stored using the EM50 data logger in the Genhe watershed. Soil permittivity (dimensionless) and temperature (°C) data were collected and stored using the XST data logger in the Saihanba area. The long-term observation data of the Genhe watershed were manually exported and stored. The observation data of the Saihanba area were transferred back to the indoor wireless network server every day.
Data source location	Genhe watershed, Inner Mongolia, China (50.16°–50.66°N, 120.5°–121°E) Saihanba area, Hebei Province, China (42°–42.5°N, 117°–117.5°E)
Data accessibility	Repository name: Mendeley Data Data identification number: http://dx.doi.org/10.17632/hj22ymt7xj.1 Direct URL to data: https://data.mendeley.com/datasets/hj22ymt7xj/1
Related research article	J. Wang, L.M. Jiang, H.Z. Cui, G.X. Wang, J.W. Yang, X.J. Liu, and X. Su, Evaluation and analysis of SMAP, AMSR2 and MEaSUREs freeze/thaw products in China. Remote Sensing of Environment, 2020. 242: p. 111734. https://doi.org/10.1016/j.rse.2020.111734.

## Value of the Data

• The dataset can provide ground truth of soil moisture and soil freeze/thaw spatial scales as evaluation or calibration of soil moisture and freeze/thaw estimates from microwave remote sensing and land hydrological modeling at regional scales.
• This dataset is beneficial to study the land surface and atmosphere interactions and climate change and water cycle on a regional scale.
• The dataset can be further used to optimize the distribution of sites by analyzing the representativeness of the data collected at those sites and to obtain high-quality observations at low cost.
• The dataset complements the existing ground observations in China.

## Data Description

The dataset contains raw data and processed data collected from the Genhe watershed and the Saihanba area. All the data are stored in a ZIP archive. The data file of each automatic observation network is named with the site name and the data level. The observed variables and data profiles at each site in the Genhe watershed and Saihanba area are shown in Table 1. There are three depth measurements (3, 5, and 10 cm) in the Genhe watershed and two (5 and 10 cm) in Saihanba. In Table 1, field names such as Soil_moisture_5 and Soil_temperature_5 mean the measurements of soil moisture and soil temperature at the depth of 5 cm below the surface in the observational network. The raw data are soil temperature and permittivity for the Saihanba area and soil moisture and soil temperature for the Genhe watershed, respectively.

**Table 1:** Automatic observation network observation items and data overview.

Field name	Column name	Data type	Dimension	Example
Measurement Time	Data acquisition time	/	/	8/29/2018 1:30 (Saihanba area, A1)
Soil_moisture_3	−3 cm soil volumetric water content	Float	m^3^/m^3^	/
Soil_temperature_3	−3 cm soil temperature	Float	°C	/
Soil_ permittivity_5	−5 cm soil permittivity	Float	/	12.7
Soil_moisture_5	−5 cm soil volumetric water content	Float	m^3^/m^3^	0.2379
Soil_temperature_5	−5 cm soil temperature	Float	°C	15.6
Soil_ permittivity_10	−10 cm soil permittivity	Float	/	15.02
Soil_moisture_10	−10 cm soil volumetric water content	Float	m^3^/m^3^	0.2761
Soil_temperature_10	−10 cm soil temperature	Float	°C	17.1

According to the sensor specifications, the accuracy of soil temperature and soil moisture observations in the Genhe watershed taken with the EC-5TM probes are 1°C and 2–3%, respectively. The accuracy of soil temperature and soil moisture observations in Saihanba area taken with the XST probes are 0.5°C and 3%, respectively. The data that could not be collected are marked as NaN. There are four sites (A8, A10, A12, and P4) with data collection failure, five sites (A2, A4, P1, P3, and P5) with data collection failure during winter, and one site (P10) with data collection failure at 10 cm depth in Saihanba.

## Experimental Design, Materials, and Methods

### Automatic observation network design and data acquisition method

**Genhe Watershed Observation Network**

The Genhe watershed has a cold and humid temperate forest climate and a continental monsoon climate. It is located in northern Inner Mongolia on the western slope of the northern Greater Khingan Range. This region has hills with gentle slopes (slopes of less than 15 degrees occupy 80% of the area) and a mean altitude of approximately 800 m. The overall geomorphology is represented by quasi-flat ground and rounded mountains with flat tops at similar altitudes [8,9]. Because of its significant geographical location, the Genhe watershed provides a representative coverage of the complex land surface and hydrometeorological conditions in Northeast China. Therefore, the *in situ* soil moisture and temperature observed network was conducted in the Genhe watershed to improve the dynamic analysis and remote sensing modeling of surface parameters, including soil moisture and surface frozen/thaw status [1,9]. In addition, the dataset would provide the surface condition to the regional biomass and carbon fluxes estimation of forest vegetation in Northeast China.

**Fig. 1:**
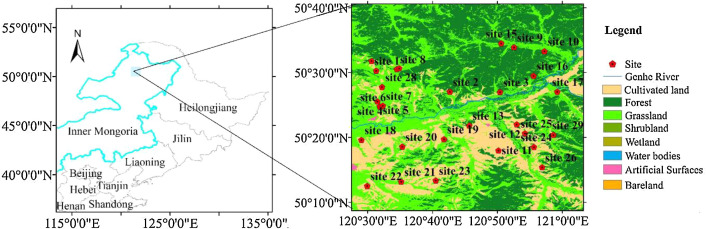
Distribution of sites in Genhe Watershed Observation Network.

The Genhe Watershed Observation Network has been operated on both sides of the Genhe watershed (50.16°–50.66°N, 120.5°–121°E) since October 2013 (7 sites), and the number of available sites was gradually increased to 22 from October 2015 to May 2017. There are four (site 1–site 3 and site 5), one (site 9), three (sites 11, 12, and 14), nine (site 15–20, 22, 24, and 26), and five (site 21, 23, 27–29) sites that have been collecting data successfully since October 2013, April 2015, October 2015, September 2016, and May 2017, respectively. Therefore, to ensure the validity of the dataset, we only provide the data with continuity and integrity and the description of observation sites from March 2016 to February 2018. The detailed information on the sites is presented in Fig. 1 and Table 2. The land cover map in Fig. 1 is from the National Geomatics Center of China (GlobeLand30-2010, http://glc30.tianditu.com).

**Table 2:** Site information of Genhe Watershed Observation Network.

Site name	Longitude (deg.)	Latitude (deg.)	Altitude (m)	Land cover	Data available time (Month/Day/Year)
Site 1	120.522	50.505	705	Grass	10/07/2013–02/28/2018
Site 2	120.711	50.451	699	Larix gmelinii	10/10/2013–02/28/2018
Site 3	120.840	50.450	628	Shrub, birch forest	10/06/2013–02/28/2018
Site 4	120.525	50.426	608	Grass, Shrub	10/07/2013–03/31/2014
Site 5	120.531	50.413	628	Grass, Shrub	10/07/2013–02/28/2018
Site 6	120.533	50.412	673	Grass	10/07/2013–10/09/2015
Site 7	120.539	50.415	792	Grass	10/07/2013–09/19/2015
Site 8	120.575	50.509	738	Birch forest	09/26/2014–04/22/2015
Site 9	120.876	50.565	705	Birch forest	04/21/2015–02/28/2018
Site 10	120.954	50.555	728	Larix gmelinii	04/21/2015–10/02/2015
Site 11	120.836	50.300	724	Shrub, Birch forest	10/10/2015–02/28/2018
Site 12	120.883	50.367	651	Shrub, Birches	10/10/2015–02/28/2018
Site 13	120.761	50.364	754	Birch forest	10/10/2015–05/10/2017
Site 14	120.581	50.511	731	Birch forest	10/09/2015–02/28/2018
Site 15	120.843	50.575	730	Larix gmelinii, Birches	09/22/2016–02/28/2018
Site 16	120.926	50.492	763	Birch forest	09/22/2016–02/28/2018
Site 17	120.987	50.451	640	Grass, Shrub	09/23/2016–02/28/2018
Site 18	120.484	50.327	608	Crop	09/24/2016–02/28/2018
Site 19	120.696	50.329	644	Shrub, Birches	09/24/2016–02/28/2018
Site 20	120.589	50.310	714	Grass, Birches	09/25/2016–02/28/2018
Site 21	120.586	50.220	731	Grass	05/14/2017–02/28/2018
Site 22	120.499	50.209	654	Crop	09/24/2016–02/28/2018
Site 23	120.675	50.223	754	Grass, Birches	05/12/2017–02/28/2018
Site 24	120.927	50.309	668	Grass	09/25/2016–02/28/2018
Site 25	120.904	50.344	681	Grass, Birches	09/25/2016–05/10/2017
Site 26	120.948	50.257	691	Grass	09/25/2016–02/28/2018
Site 27	120.510	50.530	788	Birch forest	05/09/2017–02/28/2018
Site 28	120.537	50.463	641	Grass, Shrub	05/09/2017–02/28/2018
Site 29	120.977	50.340	802	birch forest	05/15/2017–02/28/2018

The Genhe Watershed features forests, shrubland, grassland, and cultivated land. The soil texture is silt (50–54%), sand (6–9%), clay (39–44%), and organic matter (7–8%). The site of the Genhe Watershed Observation Network is equipped with the EM50 data collection system with EC-5TM probes. Soil temperature and moisture were measured every 30 min at depths of 3, 5, and 10 cm below the surface at each site. The raw data obtained using the probes are collected and stored by the data collection system, and the time series of data are stored manually from the data collection system.

**Fig. 2:**
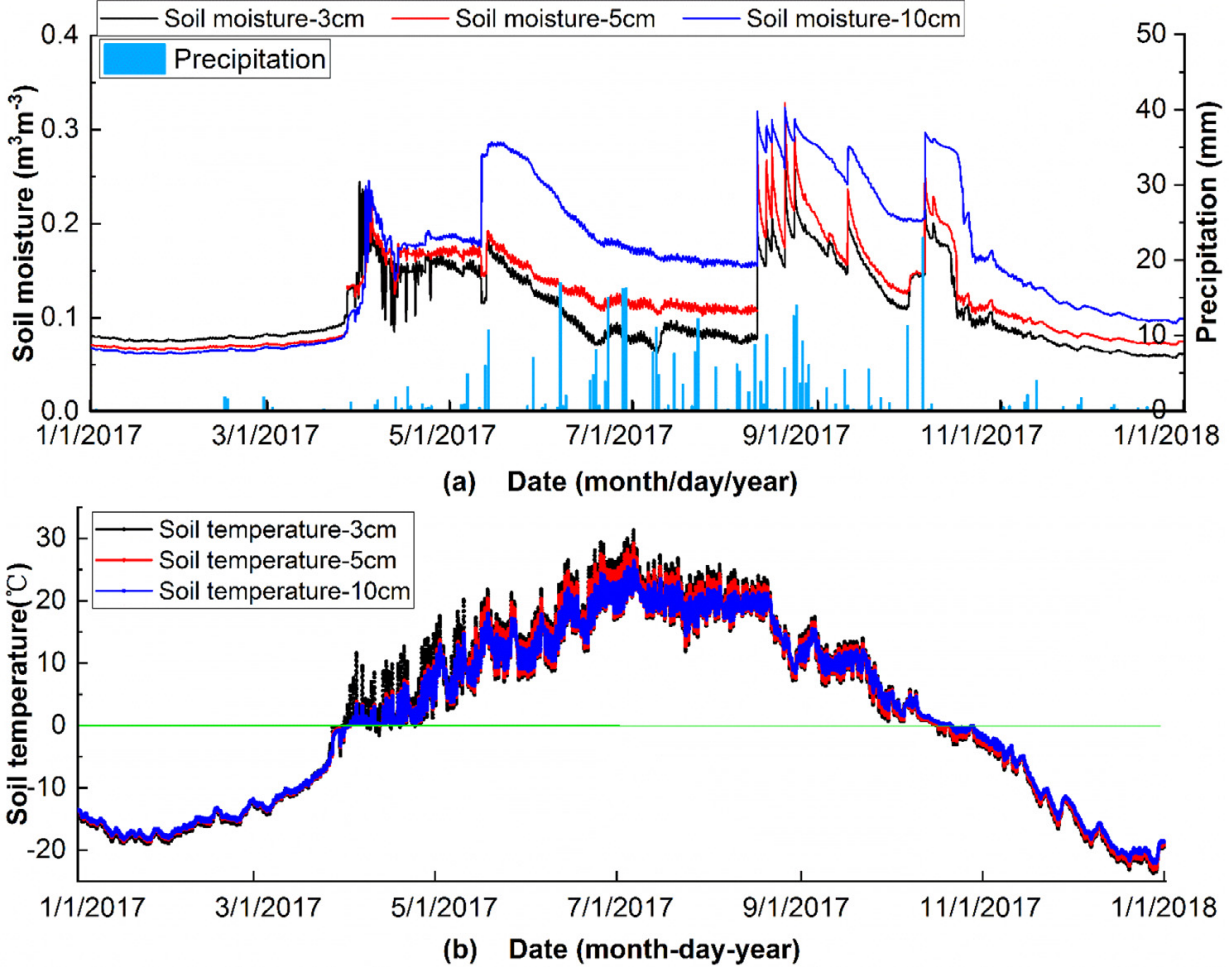
Temporal variations in observed data in the Genhe Watershed Observation Network (a: soil moisture and precipitation; b: soil temperature).

To ensure the reliability of the Genhe Watershed observation data, this work evaluated the relationship between soil moisture, soil temperature, and precipitation time series (Fig. 2) (precipitation data from the China Meteorological Data Service Center, http://data.cma.cn/). Fig. 2 (a) shows that the seasonal variations in soil moisture at depths of 3, 5, and 10 cm are similar, and the fluctuations in soil moisture at different depths within a day are not obvious. Soil moisture at three depths increases with precipitation. As the frequency of rainfall increases, soil moisture at three depths varies significantly and show an obvious stratification. Overall, the response of soil moisture to precipitation is relatively sensitive. Fig. 2 (a) shows that temporal variations in soil temperature at the depths of 3, 5, and 10 cm are similar. From January to March and from mid-October to December, surface temperature is below 0°C and the soil is frozen. When the soil is frozen, the soil moisture value remains relatively stable without significant changes. At the beginning of April, soil temperature increases above 0°C, the frozen soil begins to melt, and soil moisture gradually increases. This result is consistent with the local climate patterns. With outlier filtering, this dataset meets the requirements of data accuracy for the soil moisture retrieval algorithm development and satellite soil moisture product validation.

### Saihanba Observation Network

**Fig. 3:**
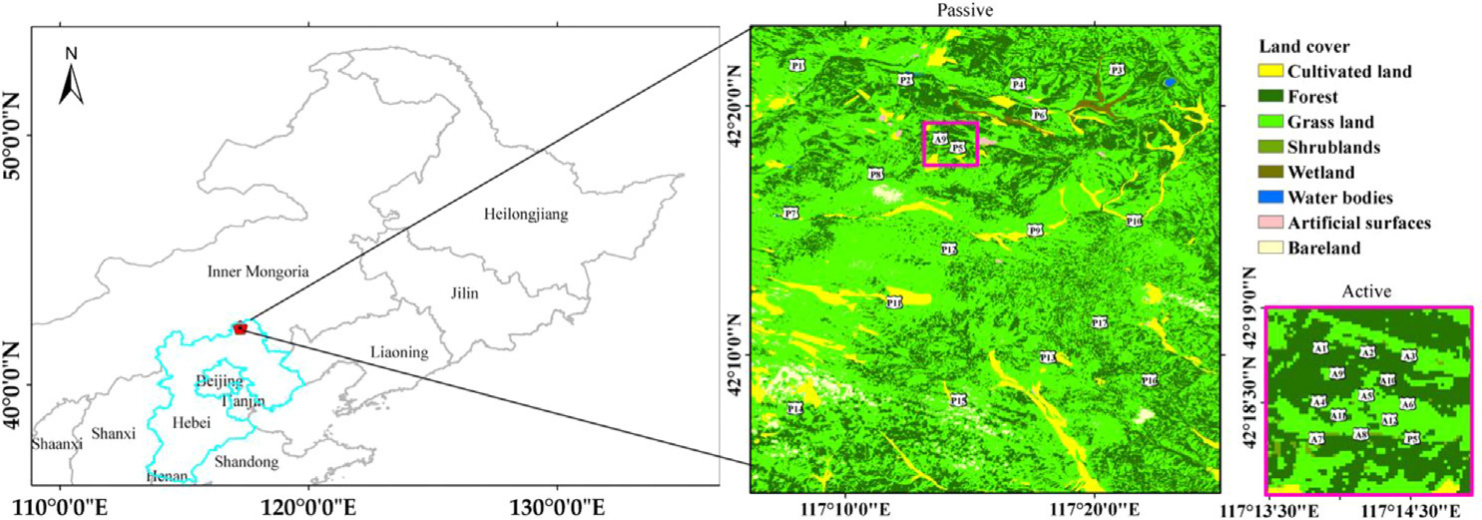
Distribution of sites in Saihanba observation network.

The Saihanba area is located in the transition zone from Yanshan Mountain to Inner Mongolia, with an elevation of 1100–1800 m and a semi-arid and semi-humid climate. As the source of the Luan River, Saihanba is a key area for global change research [10]. The soil of this area is composed of silt (12%), sand (79%), and clay (9%). With complex climate conditions and large spatial heterogeneity of soil moisture and temperature within the satellite radiometer coarse pixels of 10–50 km, sparse meteorological observation sites cannot reflect the spatial distribution of soil moisture and temperature in Saihanba. Therefore, it is important to set up a soil temperature and moisture observation network at the microwave pixel scale over the Saihanba area. The Saihanba soil temperature and moisture automatic observation network (42°–42.5°N, 117°–117.5°E) measures data both at the passive microwave pixel scale (e.g., SMAP, SMOS, AMSR2, and FY-3B) and active microwave satellite pixel scale (e.g., Sentinel-1). The observation area of the active and passive microwave pixels is 0.1° × 0.1° and 0.25° × 0.25°, respectively. There are 12 sites (named hereafter A (Active)) in active microwave pixels and 17 sites (named hereafter P (Passive)) in passive microwave pixels. The distribution of automatic observation sites is shown in Fig. 3. The land cover map is from GlobeLand30-2010 as in Fig. 1. The detailed geographical location and data availability time window at each site are shown in Table 3. Each site is equipped with the XST data collection system. In active and passive microwave pixels, XST and 5TM probes are used to measure soil temperature and permittivity at each site, respectively. One site in the passive microwave pixel (P5) also belongs to the active microwave pixel, and both XST (P5_XST) and 5TM (P5_5TM) probes are buried. At each station, two probes are used to measure soil temperature and permittivity. The two probes are horizontally inserted at 5 and 10 cm depths. The XST data loggers supplied by two dry batteries record data every 30 min and can keep working for more than one year. Same as the automatic observation network in the Genhe watershed, to prevent the rainwater damage to the data collector, the XST data loggers are sealed with a self-sealing bag. The raw soil temperature and permittivity data are measured at 5 and 10 cm depths below the surface and transformed back to the indoor server daily using a data transmission device.

**Table 3:** Site information of Saihanba observation network.

Site name	Longitude (deg.)	Latitude (deg.)	Altitude (m)	Land cover	Data available time (Month/Day/Year)
A1	117.2311	42.3131	1470	Coniferous forests	08/28/2018–02/28/2019
A2	117.2367	42.3127	1498	Grassland	08/28/2018–02/28/2019
A3	117.2416	42.3124	1520	Grassland	08/28/2018–02/28/2019
A4	117.2310	42.3084	1500	Coniferous forests	08/27/2018–02/28/2019
A5	117.2365	42.3089	1512	Grassland	08/28/2018-02/28/2019
A6	117.2414	42.3082	1522	Grassland	08/28/2018–02/28/2019
A7	117.2306	42.3051	1450	Grassland	08/27/2018–02/28/2019
A8	117.2358	42.3055	1468	Grassland	/
A9	117.2331	42.3108	1500	Coniferous forests	08/28/2018–02/28/2019
A10	117.2390	42.3102	1515	Coniferous forests	/
A11	117.2332	42.3072	1499	Coniferous forests	08/27/2018–02/28/2019
A12	117.2392	42.3067	1492	Grassland	/
P1	117.1346	42.3600	1442	Coniferous forests	09/24/2018–02/28/2019
P2	117.2070	42.3505	1454	Coniferous forests	09/24/2018–02/28/2019
P3	117.3483	42.3572	1753	Grassland	08/29/2018–02/28/2019
P4	117.2822	42.3475	1520	Grassland	/
P5	117.2419	42.3051	1498	Grassland	08/28/2018–02/28/2019
P6	117.2964	42.3269	1532	Coniferous forests	08/29/2018–02/28/2019
P7	117.1302	42.2610	1353	Grassland	08/29/2018–02/28/2019
P8	117.1870	42.2874	1428	Shrub	08/29/2018–02/28/2019
P9	117.2936	42.2495	1494	Grassland	09/23/2018–02/28/2019
P10	117.3597	42.2559	1555	Coniferous forests	09/22/2018–02/28/2019
P11	117.1994	42.2012	1436	Shrub	09/24/2018–02/28/2019
P12	117.2360	42.2369	1500	Grassland	09/23/2018–02/28/2019
P13	117.3021	42.1644	1349	Birch forest	09/23/2018–02/28/2019
P14	117.1333	42.1302	1612	Coniferous forests	09/23/2018–02/28/2019
P15	117.2423	42.1358	1314	Coniferous forests	09/23/2018–02/28/2019
P16	117.3701	42.1492	1334	Coniferous forests	09/22/2018–02/28/2019
P17	117.3367	42.1878	1639	Coniferous forests	09/22/2018–02/28/2019

**Fig. 4:**
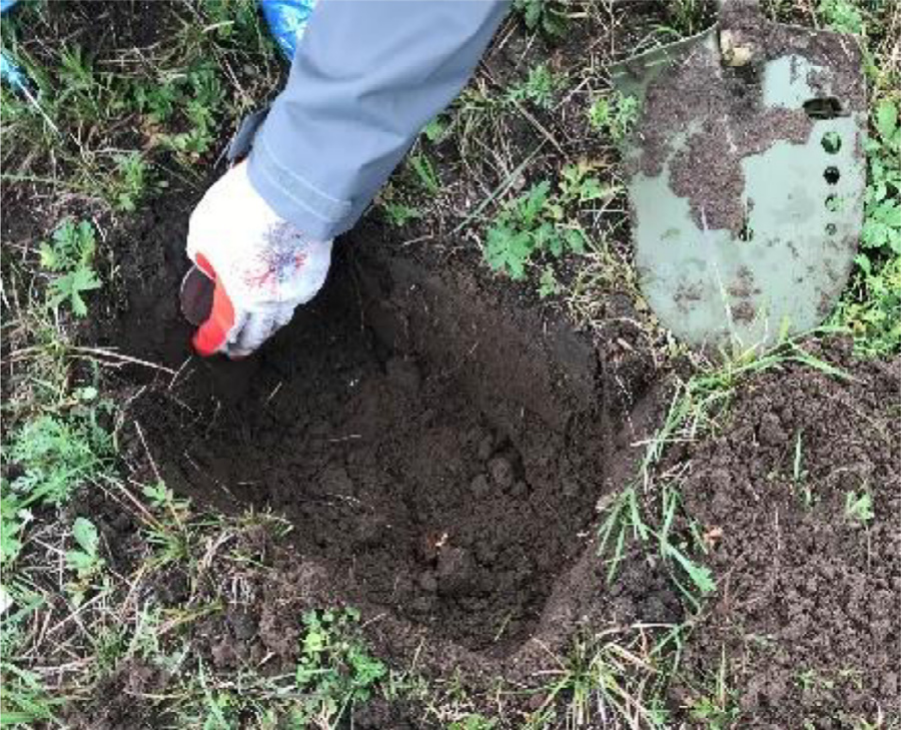
Soil sample collection with a ring cutter.

In order to ensure the authenticity of the observation data, we collected soil samples and used the soil texture data to calibrate the soil moisture data in Saihanba. The field soil samples were collected horizontally using a ring cutter with a volume of 100 mL and diameter of 5 cm (Fig. 4). The ring cutter's center corresponds to the measured depth (−5 cm, −10 cm) while collecting the soil samples. Soil permittivity data, recorded using the probes, were converted into the volumetric water content (W) using the formula developed by [7] (Eq. 1). The volumetric water content of the soil samples collected at each site was obtained in the laboratory using a drying box at 105°C for 24 h. Then, the linear relationship between the volumetric water content of soil samples and probes was used to calibrate the soil moisture observed by XST.

## Data processing

The raw data stored using the EM50 data logger included soil moisture and temperature data in the Genhe watershed, while the raw data collected with the XST data logger were soil permittivity and temperature data in the Saihanba area. Permittivity was converted to the volumetric water content (*W*) using Eq. (1).

(1) $$W=4.3\;\times \dot{10^{-6}}\cdot \varepsilon^{3}-5.5\;\times 10^{-4}\cdot \varepsilon^{2}+2.92\;\times 10^{-2}\cdot \varepsilon -5.3\;\times 10^{-2}$$

where ɛ is the measured soil permittivity.

**Fig. 5:**
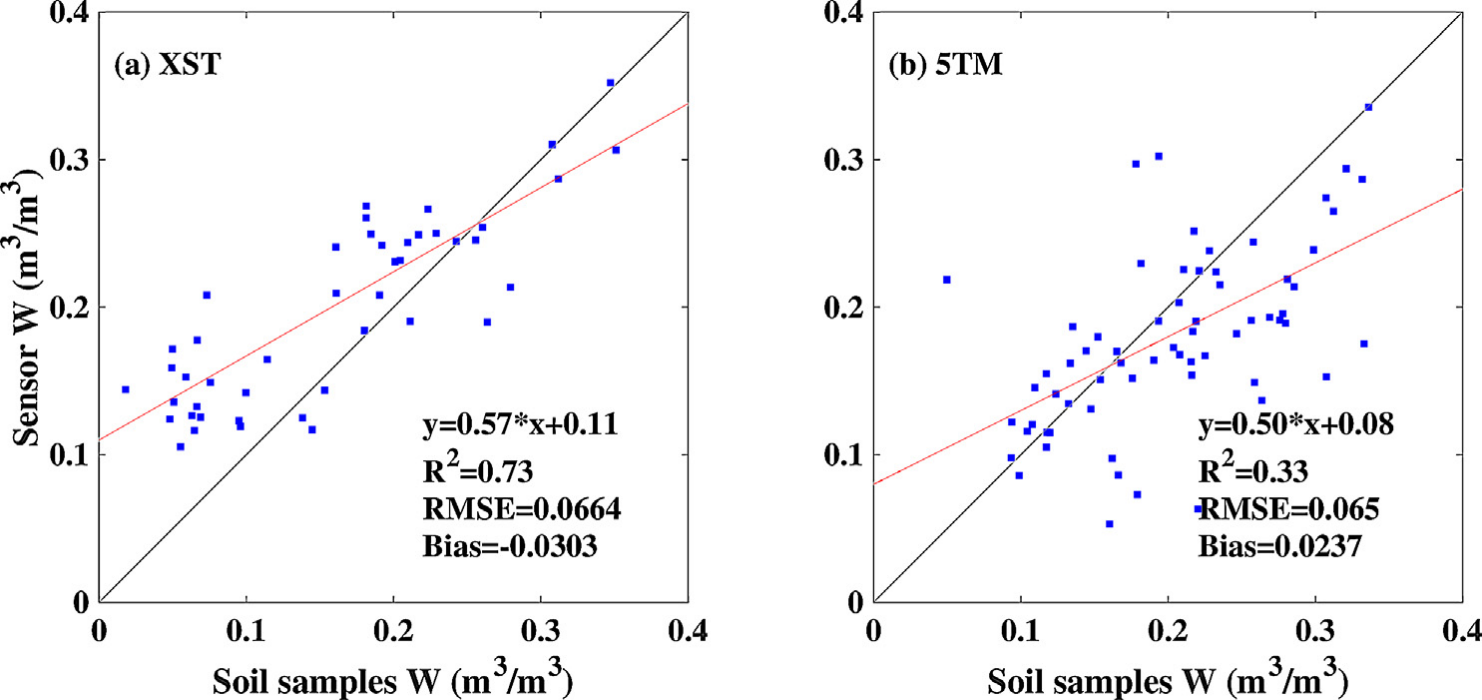
Relationship between the volumetric water content (W) calculated using soil samples and measured with the sensors over Saihanba area.

The observation data of the Genhe watershed are composed of level 0 (L0) and level 1 (L1) data, and the observation data of Saihanba are composed of L0, L1, and level 2 (L2) in the Microsoft Excel format. L0 data are the raw observation data of surface parameters taken every 30 min in the Excel format. L1 data are the valid soil temperature and moisture data taken every 30 min in the Excel format. L2 data of Saihanba are L1 soil temperature data and calibrated soil moisture data obtained by calibrating the L1 soil moisture data using the linear calibration equation in the Excel format. Fig. 5 shows the relationship between the volumetric water content calculated using the soil samples and measured with sensors over the Saihanba area.
